# Homage to Professor Osvaldo Malafaia. Editor-in-Chief of the Brazilian Archives of Digestive Surgery, ABCD from 2001 to 2021.

**DOI:** 10.1590/0102-672020210002e1611

**Published:** 2022-01-05

**Authors:** Antonio Carlos Ligocki CAMPOS, Nicolau Gregori CZECZKO

**Affiliations:** 1 Full Professor of Surgery, Department of Surgery, Parana Federal University - UFP and President-Elect of Brazilian College of Digestive Surgery - CBCD (2023-24 biennium), Brazil.; 2 Full Professor of Surgery, Department of Surgery, Parana Federal University - UFP and Mackenzie Evangelical College of Parana - FEMPAR. Ex-President of Brazilian College of Digestive Surgery - CBCD (2017-18 biennium), Brazil.; Mackenzie Evangelical College of Parana, Brazil

According to Calvin Coolidge, "persistence and determination alone are omnipotent." In the more than 45 years that we have had (and still have) the privilege of living together with Professor Osvaldo Malafaia, we believe that persistence and determination are the guidelines for his life. Always guided by ethics, knowledge, wisdom and competence, Professor Malafaia has the rare characteristic of constantly dedicating himself to his purposes. Determined to win a challenge, he doesn't know any barriers that keep him from his goals.

It was no different with the Brazilian College of Digestive Surgery (CBCD). In addition to being its President (1999-2000), when he exercised a productive administration, he was one of the creatores of the Brazilian Week of the Digestive Diseases. With the partnership of FBG and SOBED, he projected the CBCD even further in the national surgical scenario, as the main aggregating entity for Gastrintestinal Surgeons in Brazil. 

The same tenacity, persistence and determination emerged from Professor Malafaia when he accepted to be the Editor of the Brazilian Archives of Digestive Surgery, the ABCD. At the invitation of Professor Joaquim José Gama-Rodrigues (CBCD Ex-President, 2001-2002), he took on this important incumbency in 2001 ^1^. Along with Professor Nelson Adami Andreollo, over these years they elevated the ABCD to the level of the most important Journal of Surgery in Latin America, based on the indexes obtained in international organizations such as Medline/PubMed, Scientific Electronic Library Online (SciELO), Latin American and Caribbean Literature on Health Sciences - LILACS, PubMed Central - PMC and Directory of Open Access Journals - DOAJ. Its Impact Factor increased and today it stands out among Brazilian and Latin American similar magazines ^2^ ^,^ ^3^.

The journey to reach this level was arduous, the journey was difficult, but the obstacles were overcome with gallantry. Obtaining national and international recognition requires a very high level of demand. In the beginning, the journal was unknown, and quality scientific article submissions were rare and insufficient. How to identify good researchs if they were not submitted to ABCD? The idealized solution was the active search. Combined with his position as Coordinator of Medicine III at CAPES (Coordination for the Improvement of Higher Education Personnel), Professor Osvaldo saw there the opportunity to encourage, along with Postgraduate Programs in the Area of ​​Surgery, the completion of Master's and Doctoral studies in the area of ​​digestive surgery, were submitted to the periodic. It was the creative way to encourage submissions of high scientific quality articles. As a result, the level of published works improved over time. The result was the largest citation of works published in ABCD by other journals, both national and international, increasing their impact factor ^2^ ^,^ ^3^.

This is the logic that must prevail: quality of publications generates citations, which leads to an increase in submissions and, consequently, the possibility of selecting the best researchs for publication. It is important to highlight that, in addition to the Brazilian College of Digestive Surgery, the ABCD is also the official publication of the Brazilian Association of Gastric Cancer, the Brazilian Chapter of the International Hepato-Pancreato-Biliary Association and the Pancreatic Disease Study Group.

More than gratitude for the success achieved, it is essential that the national scientific community, dedicated to digestive tract surgery, recognize and perpetuate the unique role played by Professor Osvaldo Malafaia in raising the ABCD to the current scientific standard. We believe that the best way to to thank and acknowledge this herculean effort by Professor Malafaia is to continue his pioneering work, and establish the Brazilian Archives of the Digestive Surgery as the main vehicle for the dissemination of digestive surgery in our country. We wish the new responsible Editors success in this endeavor.
